# Acute bacterial prostatitis and abscess formation

**DOI:** 10.1186/s12894-016-0153-7

**Published:** 2016-07-07

**Authors:** Dong Sup Lee, Hyun-Sop Choe, Hee Youn Kim, Sun Wook Kim, Sang Rak Bae, Byung Il Yoon, Seung-Ju Lee

**Affiliations:** Department of Urology, St. Vincent’s Hospital, The Catholic University of Korea, College of Medicine, 93-6 Ji-dong Paldal-gu, Suwon, 442-723 South Korea; Department of Urology, Yeouido St. Mary’s Hospital, The Catholic University of Korea, College of Medicine, Seoul, South Korea; Department of Urology, Uijeongbu St. Mary’s Hospital, The Catholic University of Korea, College of Medicine, Uijeongbu, South Korea; Department of Urology, International St. Mary’s Hospital, Catholic Kwandong University, Incheon, South Korea

**Keywords:** Prostate, Abscess, Prostatitis, Transurethral resection of prostate

## Abstract

**Background:**

The purpose of this study was to identify risk factors for abscess formation in acute bacterial prostatitis, and to compare treatment outcomes between abscess group and non-abscess group.

**Methods:**

This is a multicenter, retrospective cohort study. All patients suspected of having an acute prostatic infection underwent computed tomography or transrectal ultrasonography to discriminate acute prostatic abscesses from acute prostatitis without abscess formation.

**Results:**

A total of 31 prostate abscesses were reviewed among 142 patients with acute prostatitis. Univariate analysis revealed that symptom duration, diabetes mellitus and voiding disturbance were predisposing factors for abscess formation in acute prostatitis. However, diabetes mellitus was not related to prostate abscess in multivariate analysis. Patients with abscesses <20 mm in size did not undergo surgery and were cured without any complications. In contrast, patients with abscesses >20 mm who underwent transurethral resection had a shorter duration of antibiotic treatment than did those who did not have surgery. Regardless of surgical treatment, both the length of hospital stay and antibiotic treatment were longer in patients with prostatic abscesses than they were in those without abscesses. However, the incidence of septic shock was not different between the two groups. A wide spectrum of microorganisms was responsible for prostate abscesses. In contrast, *Escherichia coli* was the predominant organism responsible for acute prostatitis without abscess.

**Conclusion:**

Imaging studies should be considered when patients with acute prostatitis have delayed treatment and signs of voiding disturbance. Early diagnosis is beneficial because prostatic abscesses require prolonged treatment protocols, or even require surgical drainage. Surgical drainage procedures such as transurethral resection of the prostate were not necessary in all patients with prostate abscesses. However, surgical intervention may have potential merits that reduce the antibiotic exposure period and enhance voiding function in patients with prostatic abscess.

## Background

In general, abscesses form as a result of an inflammatory process that arises from an infection. Therefore, it is reasonable to assume that a prostate abscess develops from a prostatic infection. However, to the best of our knowledge, there have been no reports comparing acute prostatitis with and without abscesses. Prostate abscess is a rare condition. Only a few reports of its diagnosis and treatment have been described over several decades [1–4]. Several groups have suggested that indwelling catheters, infravesical obstruction, instrumentation of the lower urinary tract, diabetes mellitus (DM), liver disease, and prostate biopsy are predisposing factors of prostate abscess [5, 6]. These risk factors are reasonable, but not yet supported by evidence. Patients with prostate abscess must be watched closely, as they may experience a high mortality rate if adequate and proper and timely treatment does not occur [7]. However, compared to acute prostatitis without abscess, outcomes of acute prostatitis with abscess are unclear. Here, we present data from 142 acute bacterial prostatic infections and compare acute bacterial prostatitis with and without abscess.

## Methods

### Data collection

This study is a retrospective, multicenter cohort study. Chart review for each patient was done. Distinguishable individual information was thoroughly removed during data collection. Data were gathered from four teaching hospitals with 600–1000 beds in the capital region of Korea (Seoul and Gyeonggi) using a web-based electronic system. Data were shared among all of the involved institutions. A central office managed all electronically recorded data.

### Study population and design

Any male patient between 20 and 80 years old who presented between January 2012 and December 2014 was eligible for the study. Patients were considered to have acute prostate infection if they met the following criteria: (i) one or more recent symptoms and signs such as voiding disturbance, dysuria, urgency, frequency, perineal pain, suprapubic pain or tenderness during digital rectal exam; (ii) fever (temperature of tympanic membrane >37.8 °C, [8]); and (iii) ≥5 white blood cells per high-power field in the urine. All included patients had undergone computed tomography (CT) or transrectal ultrasonography (TRUS). Such imaging studies were used to determine the presence or absence of an abscess. Successive cultures from the same patient were excluded to avoid data duplication during the treatment period. Patients with recurrent prostatitis were also excluded because the condition could negatively or positively influence susceptibility data. In order to access voiding function, uroflowmetry or post-void residual urine were checked in non-catheterized patients. Underlying diseases were reviewed based on patient medical records.

The sensitive automatic MicroScan identification system (Baxter Diagnostics, Inc., MicroScan, West Sacramento, California, USA) was used to identify causative bacteria. Minimal inhibitory concentrations (MICs) were measured using the microbroth dilution method. The following antibiotics were used to measure MICs: ampicillin, amoxicillin/clavulanic acid, cephalothin, cefoxitin, cefotaxime, ceftazidime, ciprofloxacin, imipenem, piperacillin, piperacillin/tazobactam and co-trimoxazole. A Sensitive/Intermediate/Resistant (SIR) interpretation was used for simple description and easy comparison of the data. We applied the standard Clinical and Laboratory Standards Institute guidelines to establish the MIC breakpoints.

### Statistical analysis

Student’s *t*-tests or Mann–Whitney U tests were used to compare continuous variables between the two groups. The Chi-square test or Fisher’s exact test was also used to perform univariate analysis of binominal variables. The logistic regression test was used for multivariate analysis. Each statistical method is also summarized beneath the corresponding table.

## Results

One hundred forty-two patients with acute prostatitis were enrolled, 31 of these showed prostatic abscess. Of all of the 142 patients, 101 were admitted to the urology department or department of infectious medicine from the emergency department and 41 patients were admitted to the urology department from outpatient clinics. 101 patients underwent CT scans in the emergency department. Among these 101 patients, there were 22 prostatic abscesses without other associated abscesses, two prostatic abscesses with liver abscesses, one prostatic abscess with a renal abscess, one prostatic abscess with a buttock abscess and one renal abscess without a prostatic abscess. 37 of 41 patients admitted from outpatient clinics underwent TRUS when their fever subsided. Based on this imaging study, three prostate abscesses were diagnosed. The remaining four patients underwent CT scanning because of persistent fever; this imaging diagnosed two prostate abscesses without metastatic abscess foci, and one liver abscess without a prostate abscess. Therefore, a total of 31 prostate abscesses were diagnosed and analyzed among a total of 142 acute bacterial prostatitis cases. The baseline patient demographic characteristics are summarized in Table 1.

**Table 1:** Demographic characteristics in 142 acute febrile prostatic infections

	With abscess (*n* = 31)	Without abscess (*n* = 111)	Statistical results
Underlying factors			
Age (years)†	62.23 ± 14.60	63.99 ± 14.27	0.546, 2.913, −7.524 ~ 3.994
BMI (Kg/m^2^)†	23.93 ± 4.04	24.05 ± 3.79	0.887, 0.827, −1.756 ~ 1.521
PSA (ng/ml)†	15.95 ± 11.46	20.76 ± 19.17	0.200, 3.730, −12.181 ~ 2.571
Prostate size (cc)†	47.73 ± 19.25	40.80 ± 17.53	0.063, 3.694, −0.377 ~ 14.242
Abscess size (mm)	22.03 ± 11.69	NA	
	<10 mm: 4; 10 ~ 20 mm: 12; 20 ~ 30 mm: 8; >30 mm: 7		
Other abscess foci	4; 2 liver abscesses, 1 renal abscess, 1 buttock abscess	2; 1 liver abscess, 1 renal abscess	NA
Symptom duration†	7.81 ± 5.42	3.04 ± 2.57	<0.001, 0.691, 3.404 ~ 6.136
Underlying diseases			
Diabetes mellitus‡	16/31	32/111	0.030, 2.633, 1.165 ~ 5.951
Hypertension‡	11/31	49/111	0.418, 0.696, 0.305 ~ 1.589
Heart disease‡	4/31	23/104	0.316, 0.522, 0.166 ~ 1.644
Neurological deficit‡	10/31	31/111	0.658, 1.229, 0.520 ~ 2.903
Chronic kidney disease*	4/31	16/111	1.000, 0.880, 0.271 ~ 2.851
Chronic lung disease*	2/31	11/111	0.734, 0.627, 0.131 ~ 2.991
Liver cirrhosis*	7/31	15/111	0.261, 1.867, 0.685 ~ 5.087
Previous hospitalizations‡	8/31	16/111	0.174, 2.065, 0.788 ~ 5.411
Urological procedures*	3/31 3 cystoscopy	10/111 4 cystoscopy 4 prostate biopsy 2 urodynamic study	1.000, 1.082, 0.279 ~ 4.201 0.176, 2.866, 0.606 ~ 13.553 NA NA
Long term catheterization*	2/31	12/111	0.735, 0.569, 0.120 ~ 2.689
Recent catheterization*	5/31	8/111	0.158, 2.476, 0.748 ~ 8.198
Voiding disturbance (Qmax < 5 or PVR > 100)‡	15/31	15/111	<0.001, 6.000, 2.464 ~ 14.612
Treatment outcomes			
Septic shock*	3/31	3/111	0.118, 3.857, 0.738 ~ 20.152
Death	1/31	1/111	0.390, 3.667, 0.223 ~ 60.360
Recurrence	1/31	3/111	1.000, 1.189, 0.119 ~ 11.848
Treatment duration†	40.96 ± 10.61	26.98 ± 9.43	<0.001, 2.111, 9.802 ~ 18.158
With TUR-P (*n* = 13)^a^	32.31 ± 9.34		0.001††
Without TUR-P (*n* = 13)^b^	40.85 ± 7.94		
Admission duration†	12.38 ± 6.59	6.69 ± 2.92	<0.001, 1.321, 2.978 ~ 8.403
With TUR-P (*n* = 13)^a^	10.23 ± 2.55		0.650††
Without TUR-P (*n* = 13)^b^	11.08 ± 9.32		
Follow-up duration†	216.0 ± 69.92	202.11 ± 59.01	0.302, 13.391, −12.602 ~ 40.378

†: These continuous values are expressed as means ± standard deviations and student *t*-tests were performed (statistical data include *p*-values, standard errors and 95 % confidence intervals, respectively)

‡,*: Statistical evaluation for the nominal parameters was performed using the Chi-squared test‡ or Fisher’s exact test* (statistical data include *p*-values, odds ratios and 95 % confidence intervals, respectively.)

††: *p*-values between ‘a’ and ‘b’ were evaluated using the Mann–Whitney *U* test. Note that all cases (*n* = 13) treated by TUR-P had abscesses >20 mm in size

Among the 142 cases, 6 cases with metastatic abscesses (1 patient underwent TUR-P) and 2 fatal cases were excluded when comparing the treatment duration, admission duration and follow-up duration

Abbreviations: *BMI* body mass index; *PSA* prostate specific antigen; *Qmax* (*ml*/*sec*) maximum urinary flow rate; *PVR* (*ml*) post-void residual; *TUR*-*P* transurethral resection of prostate

In this study, the symptom duration and voiding disturbance with low Qmax (<5 ml/sec) or high residual urine (>100 ml) were associated with abscess formation in acute bacterial prostatitis. Two of 17 prostate abscess patients with voiding problems had urethral stricture. The size of the prostate tended to be larger in acute prostatitis patients with abscesses than in those without abscesses, although not significantly. Over half of the patients in the abscess group (51.6 %) had DM, while 28.8 % of non-abscess patients had DM (*p* = 0.03, *OR* = 2.633). Other underlying medical diseases were not associated with the presence of prostate abscess. Catheterization also did not exert an influence on abscess formation in this study. In multivariate analysis, symptom duration and voiding disturbances were predisposing factors for abscess formation in acute prostatitis (Table 2).

**Table 2:** Multivariate analysis of risk factors for abscess formation in acute bacterial prostatitis

	*p*-value	OR	95 % CI
Voiding disturbance	0.041	2.749	1.551 ~ 12.333
Diabetes mellitus	0.153	2.064	0.764 ~ 5.575
Symptom duration	<0.001	1.343	1.166-1.548

Statistical analysis was performed using logistic regression test

**Fig. 1:**
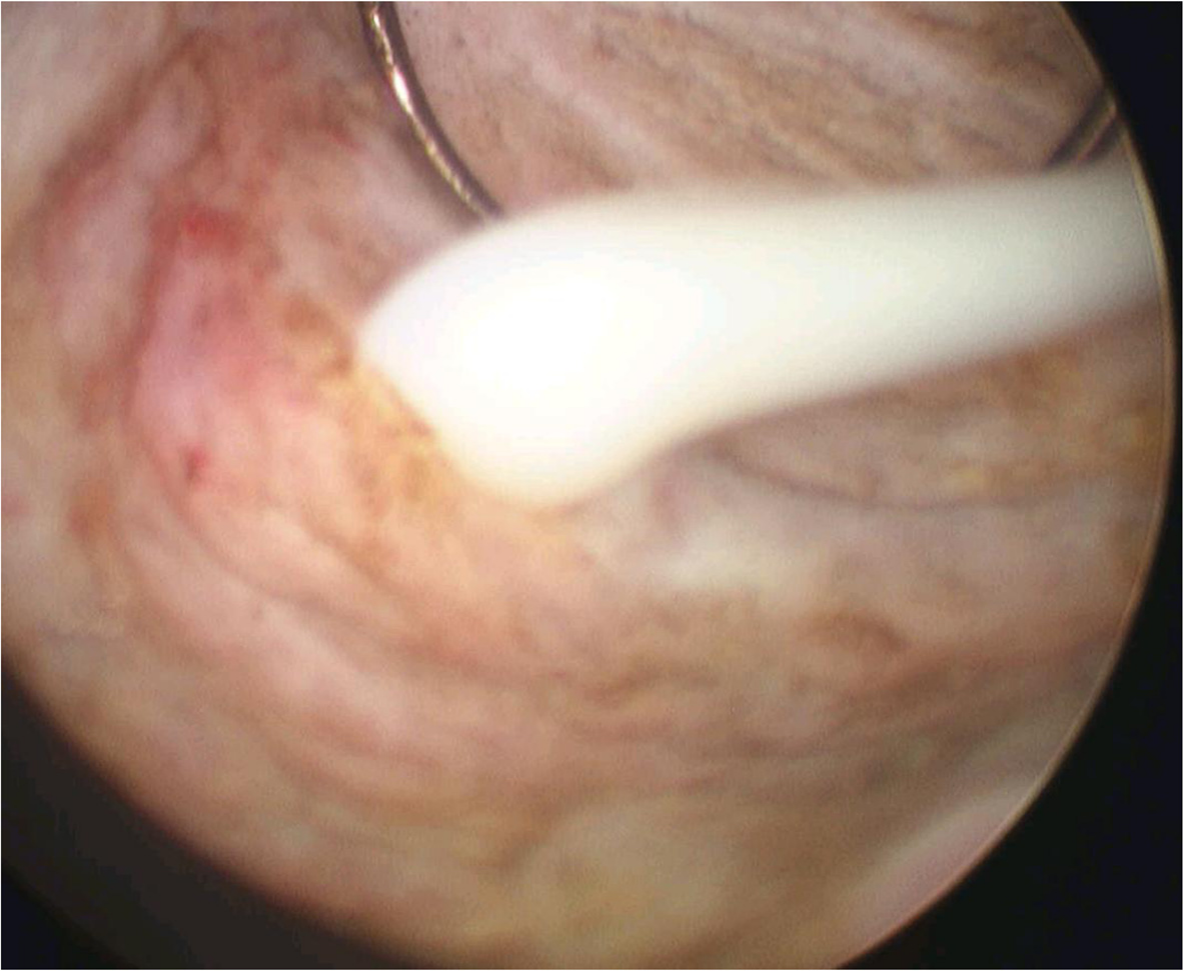
Abscess drainage with transurethral resection of the prostate

**Fig. 2:**
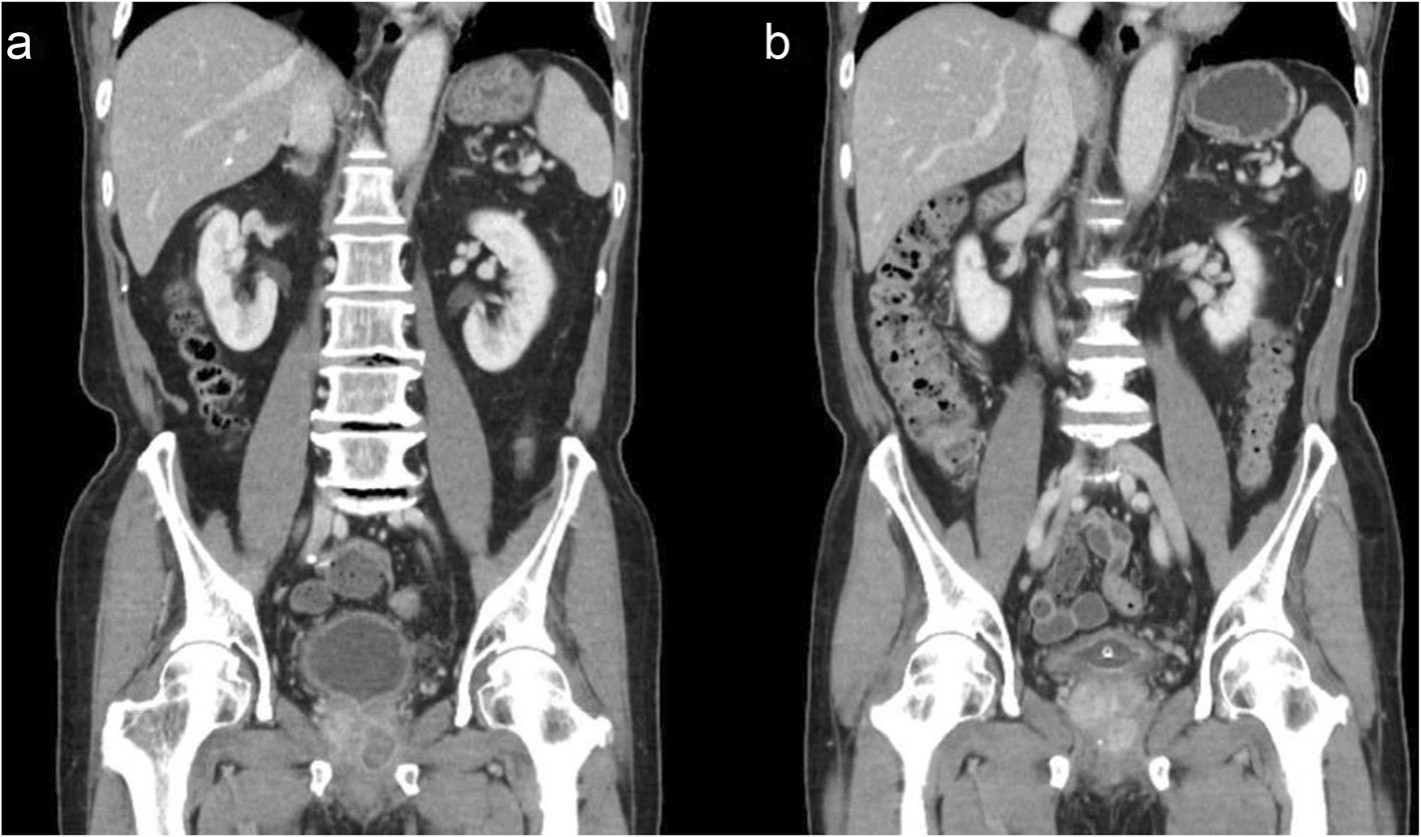
Medical treatment of prostate abscess. **a**: Before treatment (at diagnosis): an approximately 20-mm-sized abscess was identified in the left lobe of the prostate gland, **b**: After treatment (follow-up at 4 weeks): a cystostomy catheter was inserted into the urinary bladder. The previous abscess pocket was disappeared

Twenty-six patients (83.8 %) with acute prostatitis with abscesses eventually underwent suprapubic cystostomy. 14 patients with prostate abscess (45.2 %) had transurethral resection of prostate (TUR-P) during the treatment period (Fig. 1). Among those 14 patients, one with a liver abscess simultaneously underwent percutaneous drainage for liver abscess. Only one patient among 15 patients who had prostate abscess larger than 20 mm in maximal diameter, did not have any procedures, and eventually died two days after admission. 16 patients with abscesses less than 20 mm in size were treated conservatively (Fig. 2a and b). Among the 30 survivors with prostatic abscesses, only one patient who had been treated conservatively developed a recurrence within 3 months. This patient eventually underwent transurethral resection of prostate.

Six patients had additional abscess at distant sites. Among the three cases of liver abscesses, two resulted from *K. pneumoniae* infection, while the causative organism was not identified from the third. Most cases of acute prostatitis without abscess resulted from infections with *E. coli*. In contrast, various organisms were detected in cases of acute prostatitis with abscess (Table 3). S. aureus was significantly related to abscess formation (*p* = 0.03, *OR* = 12.222 by Fisher exact test). Squamous cell cancer was identified in one case of acute prostatitis with abscess.

**Table 3:** Microorganisms isolated from 142 urinary specimens

	With Abscess *n* (%)	Notes	Without abscess *n* (%)	Notes
*E. coli*	7 (26.9)	1 renal abscess	71 (74.7)	1 renal abscess, 1 death
*K. pneumoniae*	6 (23.1)	1 liver abscess	8 (8.4)	1 liver abscess
*P. aeruginosa*	4 (15.4)		4 (4.2)	
*P. mirabilis*	1 (3.8)		1 (1.1)	
*K. oxytoca*	1 (3.8)		1 (1.1)	
*Enterobacter aerogens*	1 (3.8)	1 Death	2 (2.1)	
*Enterobacter clocae*	0 (0.0)		1 (1.1)	
*Morganella morganii*	1 (3.8)		1 (1.1)	
*Serratia marcescens*	0 (0.0)		1 (1.1)	
*Citrobacter freudii*	0 (0.0)		1 (1.1)	
*S. aureus*	3 (11.5)	1 buttock abscess, 1 squamous cell carcinoma	1 (1.1)	
*E. faecalis*	1 (3.8)		1 (1.1)	
*E. faecium*	0 (0.0)		1 (1.1)	1 Death
Other GPC	1 (3.8)		1 (1.1)	
Positive culture	26		95	
Negative culture	5	1 Liver abscess	16	

The percentage of each microorganism against positive culture is presented in parentheses

In general, the treatment period with antibiotics was longer in cases of prostate abscess than in those without abscess, regardless of the surgical intervention. It is promising that patients who underwent TUR-P required shorter antibiotic treatment than did patients treated conservatively, despite the fact that the surgery group had larger prostate abscesses than did the non-surgery group (Table 1). There was no difference between prostate abscess and acute prostatitis without abscess with regard to the incidence of septic shock.

It was difficult to compare the susceptibility of *E. coli*, *K. pneumoniae* and *P. aeruginosa* between the two groups because of the small numbers of each respective isolate in abscess group. Bacteria were detected in 120 of the 142 urinary specimens. Among these, *E. coli* was isolated in 78 specimens (65.0 %), and *K. pneumoniae* in 14 cases (11.7 %). Therefore, *E. coli* and *K. pneumoniae* consisted of 76.7 % of acute prostatitis cases in this study. Antimicrobial susceptibilities (%) of the 78 *E. coli* cases to cefoxitin (second generation cephalosporins; cefamycins), cefotaxime (third-generation cephalosporins) and ciprofloxacin (fluoroquinolones) were 75/78 (96.2), 64/78 (82.1) and 63/78 (80.8), respectively. Those of the 14 *K. pneumoniae* cases to cefoxitin, cefotaxime and ciprofloxacin were 14/14 (100.0), 14/14 (100.0) and 13/14 (92.9), respectively. Antimicrobial susceptibility results in detail were presented in Table 4. The susceptibilities of *P. aeruginosa* infection to third generation cephalosporins and to fluoroquinolones were lower than 80 % in both abscess and non-abscess group. *P. aeruginosa* infection was related to previous urological procedures (by Fisher exact test, *p* = 0.002, *OR* = 13.889, 2.971 < 95 % CI < 64.927).

**Table 4:** Antimicrobial susceptibility of *E. coli*, *K. pneumoniae* and *P. aeruginosa* infections

	With abscess			Without abscess		
	*E. coli* (*n* (%))	*K. pneumoniae* (*n* (%))	*P. aeruginosa* (*n* (%))	*E. coli* (*n* (%))	*K. pneumoniae* (*n* (%))	*P. aeruginosa* (*n* (%))
Amikacin	S 7/7 (100.0)	S 6/6 (100.0)	S 3/4 (75.0)	S 71/71 (100.0)	S 8/8 (100.0)	S 4/4 (100.0)
	I 0/7	I 0/6	I 0/4	I 0/71	I 0/8	I 0/4
	R 0/7	R 0/6	R ¼	R 0/71	R 0/8	R 0/4
Amoxicillin	S 4/7 (57.1)	S 0/6 (0.0)	NA	S 31/71 (43.7)	S 0/8 (0.0)	NA
	I 0/7	I 0/6		I 0/71	I 0/8	
	R3/7	R 6/6		R 40/71	R 8/8	
Amoxicillin/clavulanic acid	S 5/7 (71.4)	S 6/6 (100.0)	NA	S 48/71 (67.6)	S 8/8 (100.0)	NA
	I 2/7	I 0/6		I 17/71	I 0/8	
	R 0/7	R 0/6		R 6/71	R 0/8	
Aztreonam	S 5/7 (71.4)	S 6/6 (100.0)	S 1/4 (25.0)	S 65/71 (91.5)	S 8/8 (100.0)	S 2/4 (50.0)
	I 0/7	I 0/6	I 1/4	I 0/74	I 0/8	I 1/4
	R 2/7	R 0/6	R 2/4	R 6/74	R 0/8	R 1/4
Cefazolin	S 5/7 (71.4)	S 6/6 (100.0)	NA	S 54/71 (76.1)	S 8/8 (100.0)	NA
	I 0/7	I 0/6		I 0/71	I 0/8	
	R 2/7	R 0/6		R 17/71	R 0/8	
Cefepime	S 5/7 (71.4)	S 6/6 (100.0)	S 3/4 (75.0)	S 67/71 (94.4)	S 8/8 (100.0)	S 3/4 (75.0)
	I 0/7	I 0/6	I 0/4	I 0/71	I 0/8	I 1/4
	R 2/7	R 0/6	R ¼	R 4/71	R 0/8	R 0/4
Cefotaxime	S 5/7 (71.4)	S 6/6 (100.0)	S 0/4 (0.0)	S 59/71 (83.1)	S 8/8 (100.0)	S 0/4 (0.0)
	I 0/7	I 0/6	I 0/4	I 0/71	I 0/8	I 0/4
	R 2/7	R 0/6	R 4/4	R 12/71	R 0/8	R 4/4
Cefoxitin	S 7/7 (100.0)	S 6/6 (100.0)	NA	S 68/71 (95.8)	S 8/8 (100.0)	NA
	I 0/0	I 0/6		I 2/71	I 0/8	
	R 0/0	R 0/6		R 1/71	R 0/8	
Ceftazidime	S 5/7 (71.4)	S 6/6 (100.0)	S 3/4 (75.0)	S 68/71 (95.8)	S 8/8 (100.0)	S 4/4 (100.0)
	I 0/7	I 0/6	I 0/4	I 0/74	I 0/8	I 0/4
	R 2/7	R 0/6	R 1/4	R 3/71	R 0/8	R 0/4
Ciprofloxacin	S 5/7 (71.4)	S 6/6 (100.0)	S 3/4 (75.0)	S 58/71 (81.7)	S 7/8 (87.5)	S 3/4 (75.0)
	I 0/7	I 0/6	I 0/4	I 0/71	I 0/8	I 0/4
	R 2/7	R 0/6	R 1/4	R 13/71	R 1/8	R 1/4
Imipenem	S 7/7 (100.0)	S 6/6 (100.0)	S 4/4 (100.0)	S 71/71 (100.0)	S 8/8 (100.0)	S 4/4 (100.0)
	I 0/0	I 0/6	I 0/4	I 0/71	I 0/8	I 0/4
	R 0/0	R 0/6	R 0/4	R 0/71	R 0/8	R 0/4
Piperacillin/tazobactam	S 7/7 (100.0)	S 6/6 (100.0)	S 2/4 (50.0)	S 68/71 (95.8)	S 7/8 (87.5)	S 4/4 (100.0)
	I 0/0	I 0/6	I 1/4	I 1/71	I 1/8	I 0/4
	R 0/0	R 0/6	R 1/4	R 2/71	R 0/8	R 0/4
Cotrimoxazole	S 5/7 (71.4)	S 6/6 (100.0)	S 0/4 (0.0)	S 56/71 (78.9)	S 6/8 (75.0)	S 0/4 (0.0)
	I 0/7	I 0/6	I 0/4	I 0/71	I 0/8	I 0/4
	R 2/7	R 0/6	R 4/4	R 15/71	R 2/8	R 4/4

Susceptible categories (S/I/R: Sensitive/Intermediate/Resistant)

## Discussion

One previous report described that approximately 6 % of prostatic abscesses develop in patients during the follow-up period after acute prostatitis [9]. However, prostatic abscess are often found in patients who do not improve with initial antibiotic therapy [6, 10]. Therefore, without routine imaging study, a prostate abscess present initially may be missed rather than developing from acute prostatitis during the follow-up period. In our 111 cases of confirmed acute prostatitis without abscess, abscess formation was not identified during the treatment period. Routine imaging studies such as CT or TRUS should be considered in cases of acute prostatitis for this reason, especially in patients with long-term symptom duration and voiding disturbances. Such imaging will allow physicians to anticipate a treatment method for an abscess, since these abscesses may require drainage [3, 7]. Ludwig et al. found that fluctuation during the digital rectal exam was present in 83.3 % of prostate abscesses. The group agreed that additional imaging is necessary to avoid missing a diagnosis of prostate abscess [2].

DM was a predisposing factor for abscess formation in univariate analysis. Studies of prostate abscesses commonly emphasize that DM is the most important predisposing medical condition [2, 3, 11]. However, diabetes by itself was not a risk factor for prostate abscess in multivariate analysis in the present study. DM is undoubtedly a serious condition that increases the risk of infection with uro-pathogens [12, 13]. However, its role in the development of prostatic abscess remains unclear and requires further investigation. Voiding disturbance was a significant risk factor for prostate abscess in the present study. Therefore, physicians should monitor voiding status in patients with acute prostatitis. In doing so, a physician can decide whether or not to perform a urinary diversion, such as suprapubic cystostomy, or to conduct imaging for the early diagnosis of a prostate abscess.

Abscess drainage with transurethral resection of prostate (TUR-P) was done in 45.2 % (14/31) of patients with prostatic abscess. The other 55.8 % of patients with abscesses only required medical treatment. We excluded confounding factors including one patient death, and 4 patients with other abscess foci when comparing the TUR-P group and medical treatment group in 31 abscesses. With regard to the length of hospital stay, it seems that medical treatment was non-inferior to surgical procedures in the treatment of prostatic abscesses. If we did not perform TUR-P in patient with prostate abscess over 20 mm, hospital stay might be longer in abscess patients. The duration of antibiotic treatment was longer in the medical treatment group (Table 1) than it was in the surgical group despite the cases treated with TUR-P had larger size of abscess pockets than the medical cases. Because TUR-P group and medical treatment group have different sizes of prostate abscess, and relevant cases in prostate abscess (*n* = 26) were small for comparison between TUR-P (*n* = 13) and medical treatment (*n* = 13), comparing treatment outcomes in 26 prostate abscesses may have potentially less clinically significant in the present study. However, considering there are wide concerns of antibiotic resistance in the community, minimizing the duration of exposure to antibiotics is an important issue. Furthermore, voiding disturbances were reflected in a large proportion of patients with prostate abscesses according to the present study. So, patients who underwent TUR-P might have an advantage. Therefore, TUR-P should be recommended to patients with prostate abscesses, although surgical procedures are not necessary for relatively small abscesses.

Regardless of the surgical procedure, the presence of a prostatic abscess did not increase the risk of septic shock during treatment. In our experience, patients with prostate abscesses require long-term antibiotic treatment and potentially surgery depending on abscess size. However, abscess formation may not exert an influence on the prognosis, such as septic shock or death, under the assumption that they were treated appropriately.

The vast majority of acute prostatitis were infected by gram negative bacteria. Regardless of the presence of an abscess, the selection of empirical antibiotics with cefoxitin, cefotaxime or ciprofloxacin would be appropriate in patients with acute prostatitis who have not undergone urological procedure (s) in Korea. Nevertheless, clinicians should be ready to adjust the antibiotic regimen according to susceptibility data, especially in the case of prostate abscess. This is because unexpected microorganisms are more likely to be isolated in acute prostatitis with abscess than in those without abscess.

This study has limitations. It is a retrospective, multi-centre chart review in design which has potential biases associated with it, however designing a randomised control trial comparing treatment outcome in 2 groups of equally matched patients with prostatic abscess is not feasible given the rarity, severity of the disease and heterogeneity of management for this condition throughout the urological community.

## Conclusions

Ulleryd et al. concluded that [14] routine radiological exams are dispensable in men with febrile urinary tract infection. However, it would be wise to perform imaging studies in patients who are suspected to have acute prostatitis when their symptom duration is relatively long, or any evidence of voiding disturbance. Acute prostatitis with abscess requires long-term antibiotic treatment, and sometimes even surgical drainage. Surgical procedures such as TUR-P in patients with prostatic abscesses are advantageous because they reduce the period of antibiotic treatment and may improve voiding symptoms. A wide range of microorganisms may be detected in prostatitis with abscess. Therefore, physicians should perform urine culture prior to administering empirical antibiotics. These cultures should be repeated, if possible, during treatment. With appropriate treatment, the prognosis of acute prostatitis with and without abscess would not differ.

## Abbreviations

CT, computed tomography; DM, diabetes mellitus; TRUS, transrectal ultrasonography; TUR-P, transurethral resection of prostate
